# Supplementary material for: Development of a Best Practice Guidance on Online Peer Support for People with Young-Onset Dementia
Source: Behav Sci (Basel). 2024 Aug 26;14(9):746. doi: 10.3390/bs14090746 (PMC11428312; doi:10.3390/bs14090746)
Supplement: Supplementary file 1 [file behavsci-14-00746-s001.zip › Supplementary File S1 - Guiding Questions.pdf]

Supplementary material File S1

Guiding questions for people to give feedback on the first draft of the Best Practice Guidance.

1) Is the Best Practice Guidance written in a way that is clear and understandable?
2) Is there anything else that we should include?
3) Is there anything that we should take out?
4) Is there anything else we could improve?
5) Should there be any changes to the format or layout? If so, what should we change?
6) What do you think of the length of the guidance? Please tick the box of your answer:

   ☐ Just right

   ☐ Too long

   ☐ Too short
7) Do you think the Best Practice Guidance would be useful for your peers / colleagues? Why (not)?
8) Is there anything else that you think is important?
