# Supplementary material for: Development of a Best Practice Guidance on Online Peer Support for People with Young-Onset Dementia
Source: Behav Sci (Basel). 2024 Aug 26;14(9):746. doi: 10.3390/bs14090746 (PMC11428312; doi:10.3390/bs14090746)
Supplement: Supplementary file 1 [file behavsci-14-00746-s001.zip › Supplementary File S2 - Best Practice Guidance (Part 1).pdf]

Guide to online peer support

For people with Young Onset Dementia

Key benefits of online peer support

- You can join from the comfort of your own home.
- You can be as much or as little involved as you want.
- There is no pressure to say or do anything if you don't want to.

This guide provides information about online peer support. It has hints and tips from people living with Young Onset Dementia. We developed this guide together with people with Young Onset Dementia, and health and social care professionals.

© Esther Loseto-Gerritzen 2024

Acknowledgements and contact information

We developed this Best Practice Guidance as part of a PhD project at the University of Nottingham between October 2019 – October 2022. This project was funded by the European Union's Horizon 2020 research and innovation program under the Marie Skłodowska-Curie Actions – Innovative Training Networks (H2020-MSCA-ITN-2018; grant agreement number: 813196).

This Best Practice Guidance is also part of the INDUCT and DISTINCT Best Practice Guidance on Human Interaction with Technology in Dementia (more information here).

The content of this Best Practice Guidance comes from:

- literature research
- focus groups with online peer support groups for people with Young Onset Dementia including 20 people with Young Onset Dementia
- an online survey filled in by 69 people with Young Onset Dementia
- interviews with 9 people with Young Onset Dementia

If you have any questions or want more information, please contact the author, Esther Loseto-Gerritzen (Esther.Loseto-Gerritzen1@nottingham.ac.uk).

This Best Practice Guidance is freely accessible and may be used by researchers, peer support facilitators and anyone with an interest in the topic. However, no part of this Best Practice Guidance may be used or reproduced without proper acknowledgement of the author.

© Esther Loseto-Gerritzen 2024

Contents

1. What different types of online peer support are there? 4
2. What can I expect from online peer support? 6
3. How can online peer support help me? 9
4. How can I overcome technological challenges? 11
5. Where can I find more information? 12

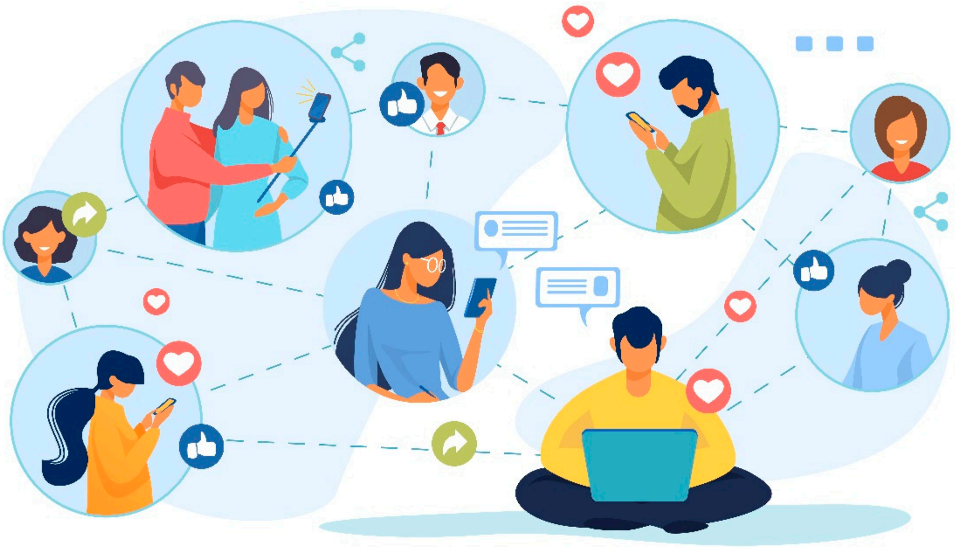

© Esther Loseto-Gerritzen 2024

1. What different types of online peer support are there?

Platforms using text and writing

- Facebook
- Twitter
- WhatsApp
- Email
- Discussion forum (for example Alzheimer Society Talking Point)

You can read and write messages whenever you want and at your own pace.

You can search for topics that are important to you.

You can't see the other people.

You don't always know the other people.

Depending on the platform, it can be a bit more anonymous. This could also be a positive.

Platforms using spoken language

- Zoom
- MS Teams
- Skype
- FaceTime

You can see the other people.

You can get to know the other people better.

You have to meet at a specific day and time.

© Esther Loseto-Gerritzen 2024

You can choose an option that works best for you. If you have difficulties with holding a conversation, a platform using text can be a good option for you. If you have difficulties reading or typing, a platform using spoken language can be a good option.

Safety first!

- Always be careful with what you share online.
- Be mindful of how online peer support affects your mental health. If you notice a negative impact, reach out to the group's facilitator or moderator. You can also take a break from it or try finding another group. You can also unfollow someone that has a negative impact.
- Always speak to your doctor before changing anything about your medication or treatment.

© Esther Loseto-Gerritzen 2024

2. What can I expect from online peer support?

Joining a new group of people can be daunting. Many people who are now part of a peer support group once felt the same. You can read about their experiences below.

What is a peer support meeting like?

- Friendly, non-judgmental group of people who are in a similar situation.
- You don't have to explain everything. The others "live in the same fog", they understand.
- It's a time to just have a chat and a laugh together.
- People share experiences and information. You can learn from others, and others can learn from you.

What could you do during a peer support meeting?

- It can be just about meeting others and having a chat.
- You may get involved in different activities. For example: music, poetry, arts and crafts.
- You can get involved in research, policy, and advocacy.

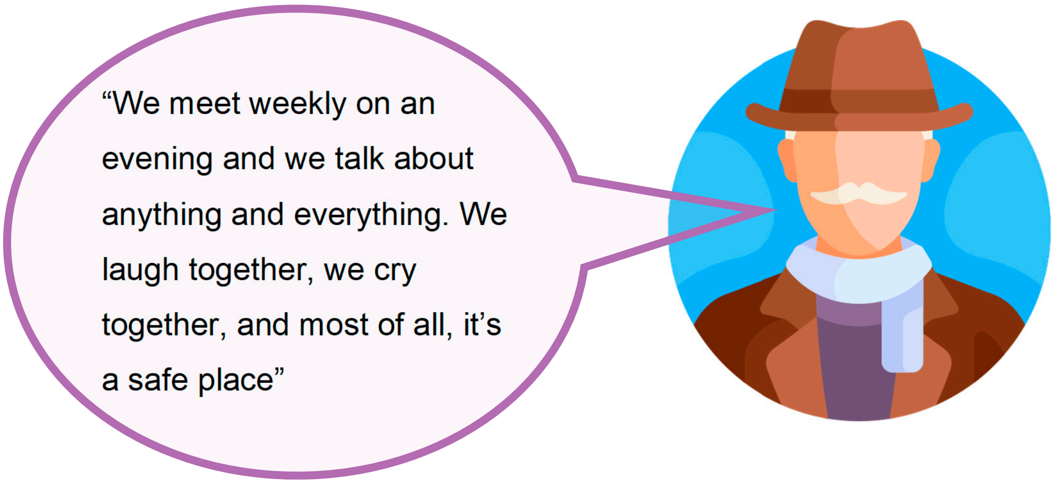

© Esther Loseto-Gerritzen 2024

What if I don’t know what to say?

You don’t have to say anything if you don’t want to. It’s ok to just listen and observe. If you are in a Zoom meeting, you can also mute your microphone or turn off your camera.

Do I have to join every meeting?

No, you don’t have to join every meeting.

What if it’s not for me?

- Finding the right group can take some trial and error. Don’t give up if the first group doesn’t suit you. There might be another one that does.
- You may try different types of online peer support and see what you prefer. For example, Zoom meetings and Facebook groups.
- **You are not alone**. There are others out there who are going through something similar.

“I was silent for quite a long time when we started because (a) I didn’t know what to say, and (b) I didn’t really want to be there, I was kind of in denial with everything. But gradually I thought ‘actually this is alright’. It’s like with any sort of introduction to anybody, it takes a little while to get in there, but it’s definitely worth it”

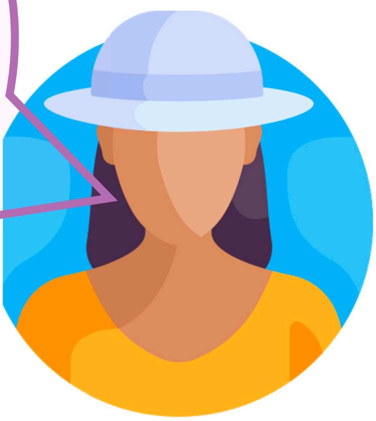

© Esther Loseto-Gerritzen 2024

What does the facilitator or moderator do?

A facilitator is there in for example video meetings. This person could be a professional, someone living with dementia, or someone who supports a person with dementia. The facilitator:

- Sends out the link for the meeting.
- Supports the meeting and steps in if necessary.
- Makes sure everyone gets a chance to speak.
- Is there for you if you have any questions.

A moderator is there in for example Facebook groups or discussion forums. This person could be a professional, someone living with dementia, or someone who supports a person with dementia. This person:

- Makes sure that the group is a safe space for everyone. For example, they delete harmful or inappropriate posts.
- Could introduce a new topic or ask a question and invite everyone to respond.
- Is there for you if you have questions.

Key points

- Peer support is friendly and non-judgmental.
- You don’t have to say anything if you don’t want to. It’s ok to just observe.
- Different groups can have different goals. Find a group that matches your needs and preferences.

© Esther Loseto-Gerritzen 2024

3. How can online peer support help me?

What can I get out of it?

- Meet new people who might have similar experiences.
- Learn about support and information resources.
- Learn from other people's experiences. They may have gone through things that you haven't (yet).
- Share your experiences. Other people can learn from you too!

"We're all like in the same room together, but without all that stimulation that you have when you're in a room with nine other people. Zoom is just such a blessing".

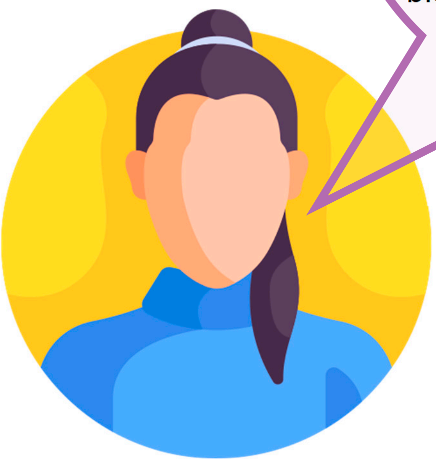

© Esther Loseto-Gerritzen 2024

What is the advantage of <u>online</u> peer support?

- There may not be anyone else in your area who is around the same age.
- There may not be anyone else in your area with the same diagnosis.
- Online you can meet people from all over the country and the world.
- You don't have to travel anywhere.
- You can join from the comfort of your own home.
- You can turn off your camera, mute yourself, or leave the meeting at any time.

"I've had some struggle to get out. Even if it was a local meeting I'd possibly have had problems actually physically getting there. Being able to have this, it breaks down lots of barriers because of distance, we can meet wherever we are internationally, but also if you struggle mobility wise or with anxiety about getting places, that sort of thing, it takes that away."

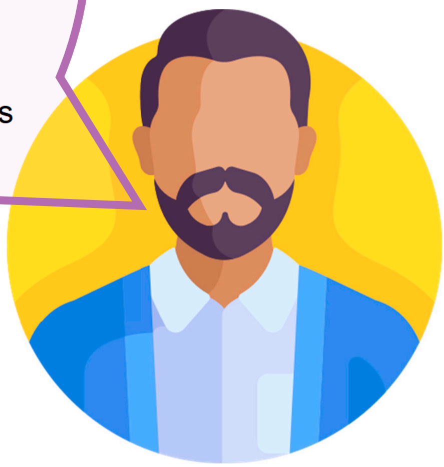

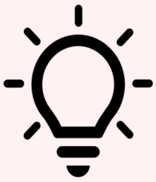

Did you know?

Even just listening to or reading about other people's experiences can be very helpful and make you feel less alone in your experiences.

© Esther Loseto-Gerritzen 2024

4. How can I overcome technological challenges?

Everyone can have trouble with technology sometimes. Below you can read about some of the challenges that some people with Young Onset Dementia face and how they overcome these.

I can't get in the Zoom meeting. What can I do?

- Contact someone else from the group or the group facilitator. They can send you the link again and help you to get in.
- Check the internet connection.
- Tip: try to get in 10 minutes before the meeting starts. This will give you enough time in case something goes wrong.

My dementia makes it difficult to use technology. What can I do?

- If you experience sensory overload, it can help to try to focus on one sense at the time.
  - If you are reading, cover your ears so you don't get distracted by background noise.
  - If you are in a video meeting, close your eyes so you can focus on listening to what the others are saying.
- Tell the group facilitator about your symptoms and what challenges you face. They can consider this during the meeting and make things as smooth as possible for you.

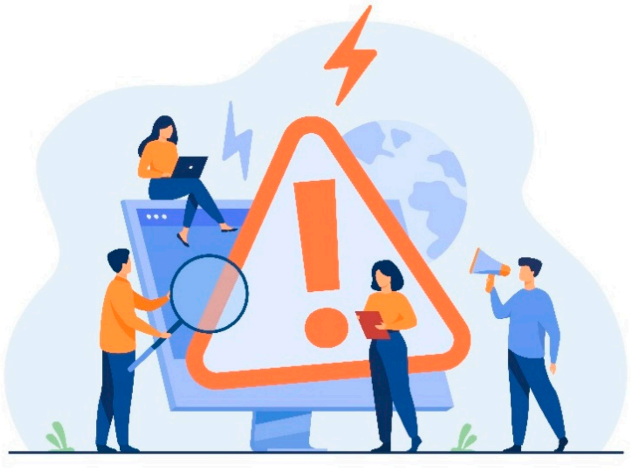

© Esther Loseto-Gerritzen 2024

5. Where can I find more information?

I want to find a peer support group.

1. DEEP (Dementia Engagement and Empowerment Project)

DEEP is a UK-wide network for peer support for people with dementia. You can find online groups and groups in your local area.

- Website: www.dementiavoices.org.uk
- Email: Rachel Niblock, coordinator. niblock@myid.org.uk
- Phone: 07720 538851

2. Rare Dementia Support

Rare Dementia Support (RDS) is a service provided by University College London (UCL). They offer information and support for people affected by a rare type of dementia.

- Website: www.raredementiasupport.org
- Email: contact@raredementiasupport.org (for the specialist support team) OR r.mckee-jackson@ucl.ac.uk (for information about support groups)

3. Opening Doors

Opening Doors is a UK charity that offers activities, events, information, and support for people from the LGBTQ+ community who are over 50. They also have monthly peer support meetings via Zoom for people living with dementia.

- Website: www.openingdoors.lgbt/ (click here for peer support information)
- Email: info@openingdoors.lgbt OR click here for the contact form on the website
- Phone: 0207 183 6260

Continued on the next page

© Esther Loseto-Gerritzen 2024

4. Dementia Alliance International

Dementia Alliance International (DAI) provides information and connects people with dementia from all over the world. They have a Facebook group and weekly peer support meetings via Zoom. These are for people with a diagnosis only.

- Website: www.dementiaallianceinternational.org (click here for peer support information)
- Contact them by filling in the contact form on the website.

© Esther Loseto-Gerritzen 2024

I want to learn from other people's experiences, but not be part of a group.

1. Dementia Diaries

Dementia Diaries brings together people's experiences of living with dementia through audio diaries. You can listen to the audio diaries at any time.

- Website: www.dementiadiaries.org
- Email: Steve Milton, coordinator. steve@myid.org.uk. You can also fill out the contact form on the website.
- Phone: 07549 944795

2. Blogs

Many people with Young Onset Dementia write about their experiences in a blog. You can find an overview of different blogs here.

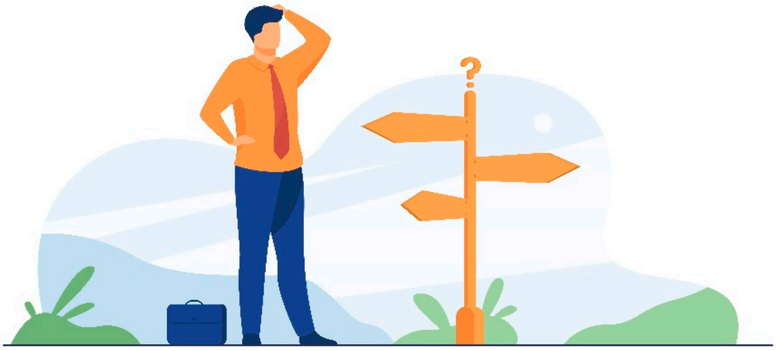

© Esther Loseto-Gerritzen 2024

I want to more information about Young Onset Dementia.

1. Young Dementia Network

This network consists of people living with Young Onset Dementia, their families, and professionals to improve the lives of people affected with Young Onset Dementia. Here you can also find more information about research and advocacy.

- Website: www.youngdementianetwork.org
- Email: youngdementianetwork@dementiauk.org

2. Dementia UK

Dementia UK is a specialist dementia nurse charity. Here you can also find specialist information about Young Onset Dementia and peer support.

- Website: www.dementiauk.org/about-dementia/young-onset-dementia/
- Email: info@dementiauk.org
- Phone: 020 8036 5400

3. Alzheimer's Society

Alzheimer's Society provides information and support services for people affected by dementia. They also have specialised information on Young Onset Dementia and support services.

- Website: www.alzheimers.org.uk/about-dementia/types-dementia/young-onset-dementia#content-start (general website) OR www.dementiaconnect.alzheimers.org.uk (support)
- Phone: 0330 333 0804 (general questions) OR 0333 150 3456 (Dementia Connect support line)

© Esther Loseto-Gerritzen 2024
