# Supplementary material for: Development of a Best Practice Guidance on Online Peer Support for People with Young-Onset Dementia
Source: Behav Sci (Basel). 2024 Aug 26;14(9):746. doi: 10.3390/bs14090746 (PMC11428312; doi:10.3390/bs14090746)
Supplement: Supplementary file 1 [file behavsci-14-00746-s001.zip › Supplementary File S3 - Best Practice Guidance (Part 2).pdf]

Online peer support for people with Young Onset Dementia

A guide for facilitators

Key messages

- Get to know people's expectations, needs, and wishes before they join.
- Timely reminders and (technological) support are important for peer support through video meetings.
- When using text-based platforms, make sure it is for members only, and have a statement on the purpose of the group.

This guide includes practical hints and tips on facilitating online peer support for people with Young Onset Dementia. It includes information on facilitating through video meetings as well as text-based platforms. We developed this guide together with people with Young Onset Dementia, facilitators, and health and social care professionals.

© Esther Loseto-Gerritzen 2024

Acknowledgements and contact information

We developed this Best Practice Guidance as part of a PhD project at the University of Nottingham between October 2019 – October 2022. This project was funded by the European Union's Horizon 2020 research and innovation program under the Marie Skłodowska-Curie Actions – Innovative Training Networks (H2020-MSCA-ITN-2018; grant agreement number: 813196).

This Best Practice Guidance is also part of the INDUCT and DISTINCT Best Practice Guidance on Human Interaction with Technology in Dementia (more information here).

The content of this Best Practice Guidance comes from:

- literature research
- focus groups with online peer support groups for people with Young Onset Dementia including 20 people with Young Onset Dementia
- an online survey filled in by 69 people with Young Onset Dementia
- interviews with 9 people with Young Onset Dementia

If you have any questions or want more information, please contact the author, Esther Gerritzen (Esther.Loseto-Gerritzen1@nottingham.ac.uk).

This Best Practice Guidance is freely accessible and may be used by researchers, peer support facilitators and anyone with an interest in the topic. However, no part of this Best Practice Guidance may be used or reproduced without proper acknowledgement of the author.

© Esther Loseto-Gerritzen 2024

Contents

Peer support in video meetings 4
1. What is important before the meeting? 4
2. What is important during the meeting? 6
3. What is important after the meeting 7
4. Further practical things to keep in mind 8
Peer support in text-based platforms 9

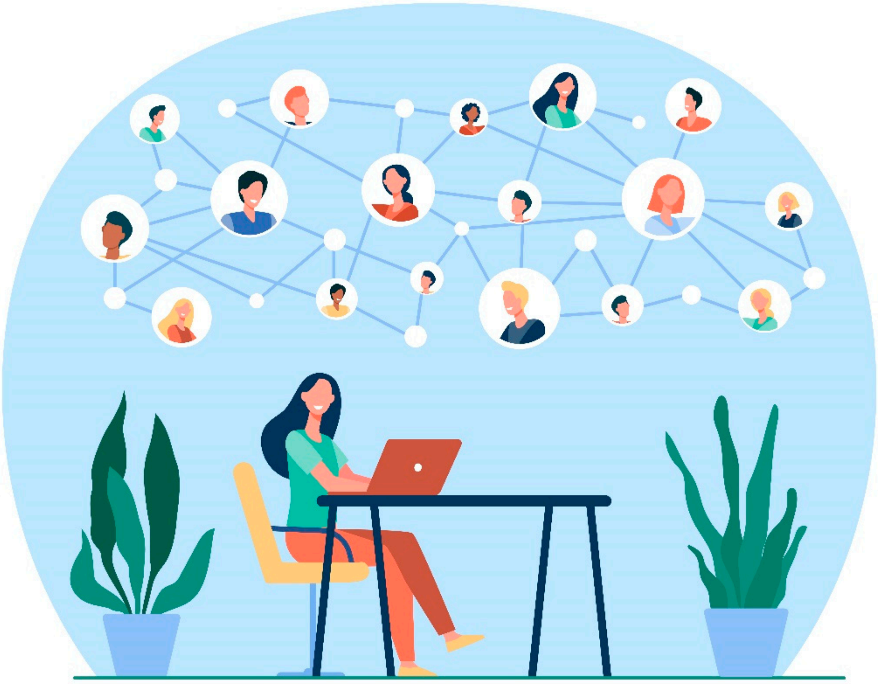

© Esther Loseto-Gerritzen 2024

Peer support in video meetings

1. What is important before the meeting?

Get to know the person. Outline whom the group is for and check with them.

- Find out if the person has similarities with the other group members. Sharing similarities can make people feel more comfortable to share things, and feel more connected.
- Only having Young Onset Dementia in common might not be enough. Other things, for example age, time since diagnosis, and dementia-subtype can be important as well. In addition, non-dementia related things, such as hobbies, interests, or professional background can also increase feelings of similarity.
- Identify what someone expects from peer support and what they want to get out of it, and whether this matches with the group.
- Identify someone's needs in terms of (technological) support.

Ground rules. Establish ground rules together with the group and regular revisit and revise them. Below are some suggestions for ground rules:

- Respect people's privacy. Things that people share in the group are confidential and should not be shared with others.
- If someone wants to take a photo or a screenshot of the meeting, ask others first, so that people can turn off their camera or leave if they don't want to be in the picture.
- Mute yourself when you're not speaking to reduce echo and background noise.
- If someone wants to say something, they should raise their hand or hold op the yellow 'I want to speak please' card.

© Esther Loseto-Gerritzen 2024

Other practical things

- Send out timely reminders, well in advance, but also closer to the day of the meeting (preferably the day before and on the day itself as well). Include the meeting link.
- Provide a guide on how to use the meeting platform.
- Be available to provide (technical) support.
- Open the meeting 10-15 minutes before the official start time, to allow people to come in and have a chat.
- Remind people that it's ok if they just want to listen in and observe.
- Remind people that if they need to step out of the meeting or need to turn off their camera at any point, they can.
- If it's a new group, or if new members are joining, prepare an ice breaker activity and allow enough time for introductions and sharing one's story.

Key points

- Get to know the person well before they join the group. Find out what their expectations, needs, and wishes are.
- Establish some ground rules with the group and repeat and revise these regularly.
- Send out timely reminders and be available to provide support.

© Esther Loseto-Gerritzen 2024

2. What is important during the meeting?

- Give everyone a chance to speak and don't let one person dominate the conversation.
- Remember who raised their hand/card first, and make sure to address everyone in order.
- Allow the group to share what they feel is important. Even if there's an agenda for the meeting, sometimes there are more important matters that people want to discuss, for example something impactful that happened in their lives.
- If you use an agenda, ask the group what items they would like to be on it.

Key points

- Give everyone a chance to speak.
- Allow the group to discuss what is important to them (either through an agenda or on the spot).

© Esther Loseto-Gerritzen 2024

3. What is important after the meeting

- If someone appears to be distressed during the meeting, or leaves the meeting abruptly, check in with them afterwards.
- Depending on the context and nature of the meeting, share some notes. For example, if it was a meeting related to research, advocacy, or policy, it can be helpful for members to have some notes to refer back to.
- Follow up on any unanswered questions that came up during the meeting.

Key points

- Check in with people afterwards if they left the meeting suddenly or appeared distressed.
- Follow up with any notes or answers to questions.

© Esther Loseto-Gerritzen 2024

4. Further practical things to keep in mind

- Group size should be limited to 10-12 people maximum.
- Duration of the meeting should be no longer than 1.5 hour.
- Try to offer meetings on different days of the week and different times of the day, to accommodate to different schedules and needs. For example, if the meeting is always on the same date (e.g. every 1st of the month), it will be on a different day every month.
- Ask the group how they feel about the chat function. Some people may find it distracting if in the middle of the meeting chats pop up, however, some may find it helpful.
- Many people prefer Zoom over other platforms such as MS Teams, as it's more user friendly and widely used.

Key points

- Max. 10-12 people.
- Max 1.5 hour.
- Offer meeting on different times and days of the week.

© Esther Loseto-Gerritzen 2024

Peer support in text-based platforms

There are different text-based platforms that can be used for peer support. For example:

- Facebook
- WhatsApp
- Discussion forums.

Particularly Facebook groups and discussion forums allow for a large membership. There are many different needs and expectations from the platform. Some may want to find more information, others may want to make new social connections and build friendships. It's important to tailor towards different needs and wishes within the platform.

Purpose of the group

- Be clear who the group is for and what the purpose is. For example, is it for people with a dementia diagnosis only, or is it also for carers?
- Provide a clear statement of the purpose of the group. This should be pinned at the home page, or be send to new members before joining the group. The statement should include:
    - Who the group is for;
    - What kind of content will not be allowed (e.g. advertisements for drugs or offensive and inappropriate messages);
    - Who the moderators are, their (professional) background, and their role;
    - A 'safety first' reminder. People should always be careful with what information they share online, and they should always speak to their doctor regarding any medication or treatment.
    - to respect other members' privacy.
- Make sure the group is a closed group, and that you as a moderator need to allow someone to join. In this way, you reduce the risk that people join the group for whom it is not really intended.

© Esther Loseto-Gerritzen 2024

Your role as a moderator / facilitator

- Monitor content on a regular basis and remove posts that are not allowed. If someone shares inappropriate content, contact this person privately (if the platform allows this).
- If someone is not receiving a response to a message, try to bring this post to the front/top of the page, invite others to respond, or provide a response from a moderator perspective.
- As a moderator you could start a discussion topic and invite members to respond. You could do a poll among members to get an insight into which topics are important for them.
- The discussion topic could be related to dementia symptoms or support services, but it could also relate to living well with dementia or hobbies and other interests.
- Based on the discussion topic, you could set up smaller 'rooms' or groups within the platform. This allows people to find a topic that suits their needs and interests and search the history on relevant topics.
- Depending on the platform and the purpose of the group, you could schedule a Q&A session with a professional. For example, concerns around driving and dementia, or lasting power of attorney. For such topics people might find it helpful to ask their questions directly to a professional.

Key points

- Make a clear statement on purpose of the group and whom it's for.
- Make the group closed so that you as a moderator need to give permission for people to join (to ensure that only people join whom the group is actually for).
- Welcome new members.
- Monitor content, remove inappropriate content, and if possible contact the author of inappropriate content.
- Create a dedicated space to safe resources and discussion topics (weather this is feasible depends on the platform).

© Esther Loseto-Gerritzen 2024
